# A Machine Learning–Based Algorithm for the Prediction of Intensive Care Unit Delirium (PRIDE): Retrospective Study

**DOI:** 10.2196/23401

**Published:** 2021-07-26

**Authors:** Sujeong Hur, Ryoung-Eun Ko, Junsang Yoo, Juhyung Ha, Won Chul Cha, Chi Ryang Chung

**Affiliations:** 1 Department of Patient Experience Management Part Samsung Medical Center Seoul Republic of Korea; 2 Department of Digital Health SAIHST Sungkyunkwan University Seoul Republic of Korea; 3 Department of Critical Care Medicine and Medicine Samsung Medical Center Sungkyunkwan University School of Medicine Seoul Republic of Korea; 4 Department of Nursing College of Nursing Sahmyook University Seoul Republic of Korea; 5 Department of Computer Science Indiana University Bloomington, IN United States; 6 Department of Emergency Medicine Samsung Medical Center Sungkyunkwan University School of Medicine Seoul Republic of Korea; 7 Digital Innovation Center Samsung Medical Center Seoul Republic of Korea

**Keywords:** clinical prediction, delirium, electronic health record, intensive care unit, machine learning

## Abstract

**Background:**

Delirium frequently occurs among patients admitted to the intensive care unit (ICU). There is limited evidence to support interventions to treat or resolve delirium in patients who have already developed delirium. Therefore, the early recognition and prevention of delirium are important in the management of critically ill patients.

**Objective:**

This study aims to develop and validate a delirium prediction model within 24 hours of admission to the ICU using electronic health record data. The algorithm was named the Prediction of ICU Delirium (PRIDE).

**Methods:**

This is a retrospective cohort study performed at a tertiary referral hospital with 120 ICU beds. We only included patients who were 18 years or older at the time of admission and who stayed in the medical or surgical ICU. Patients were excluded if they lacked a Confusion Assessment Method for the ICU record from the day of ICU admission or if they had a positive Confusion Assessment Method for the ICU record at the time of ICU admission. The algorithm to predict delirium was developed using patient data from the first 2 years of the study period and validated using patient data from the last 6 months. Random forest (RF), Extreme Gradient Boosting (XGBoost), deep neural network (DNN), and logistic regression (LR) were used. The algorithms were externally validated using MIMIC-III data, and the algorithm with the largest area under the receiver operating characteristics (AUROC) curve in the external data set was named the PRIDE algorithm.

**Results:**

A total of 37,543 cases were collected. After patient exclusion, 12,409 remained as our study population, of which 3816 (30.8%) patients experienced delirium incidents during the study period. Based on the exclusion criteria, out of the 96,016 ICU admission cases in the MIMIC-III data set, 2061 cases were included, and 272 (13.2%) delirium incidents occurred. The average AUROCs and 95% CIs for internal validation were 0.916 (95% CI 0.916-0.916) for RF, 0.919 (95% CI 0.919-0.919) for XGBoost, 0.881 (95% CI 0.878-0.884) for DNN, and 0.875 (95% CI 0.875-0.875) for LR. Regarding the external validation, the best AUROC were 0.721 (95% CI 0.72-0.721) for RF, 0.697 (95% CI 0.695-0.699) for XGBoost, 0.655 (95% CI 0.654-0.657) for DNN, and 0.631 (95% CI 0.631-0.631) for LR. The Brier score of the RF model is 0.168, indicating that it is well-calibrated.

**Conclusions:**

A machine learning approach based on electronic health record data can be used to predict delirium within 24 hours of ICU admission. RF, XGBoost, DNN, and LR models were used, and they effectively predicted delirium. However, with the potential to advise ICU physicians and prevent ICU delirium, prospective studies are required to verify the algorithm’s performance.

## Introduction

Delirium, defined as acute brain dysfunction characterized by disturbances of awareness, attention, and cognition with a fluctuating course linked with an underlying medical condition, frequently occurs among patients admitted to intensive care units (ICUs) [1]. Up to 80% of critically ill patients affected by delirium are at an increased risk of requiring ventilation for a substantially long duration, high hospital and ICU mortality, and long-term cognitive impairment. The medical care for these patients also results in increased medical costs [2-4].

There is currently limited evidence to support interventions to treat or resolve delirium in patients who have already developed delirium [5]. Therefore, the early recognition and prevention of delirium are indispensable for patients with a high risk of developing delirium. Previous studies have shown that a proportion of the cases of delirium may be avoidable [6]. Accordingly, several prediction models have been developed to predict delirium in patients who may benefit from delirium prevention [7-9]. The models developed thus far focus on predicting delirium during the entire ICU stay using predisposing clinical features obtained within 24 hours of ICU admission or immediately upon ICU admission. Considering that ICU patients experience dynamic changes in medical conditions within the initial 24 hours after ICU admission, these models are limited because they focus on predicting only the long-term occurrence of delirium during the entire ICU stay. Furthermore, these prediction models only include variables that have already been identified as risk factors for delirium in other studies [7,9,10].

Therefore, we developed a machine learning–based model for the early prediction of delirium among medical and surgical ICU patients using electronic health record (EHR) data. This prediction model uses data obtained within 4 hours of ICU admission to predict delirium within 24 hours after ICU admission.

## Methods

### Study Setting and Population

**Figure 1:**
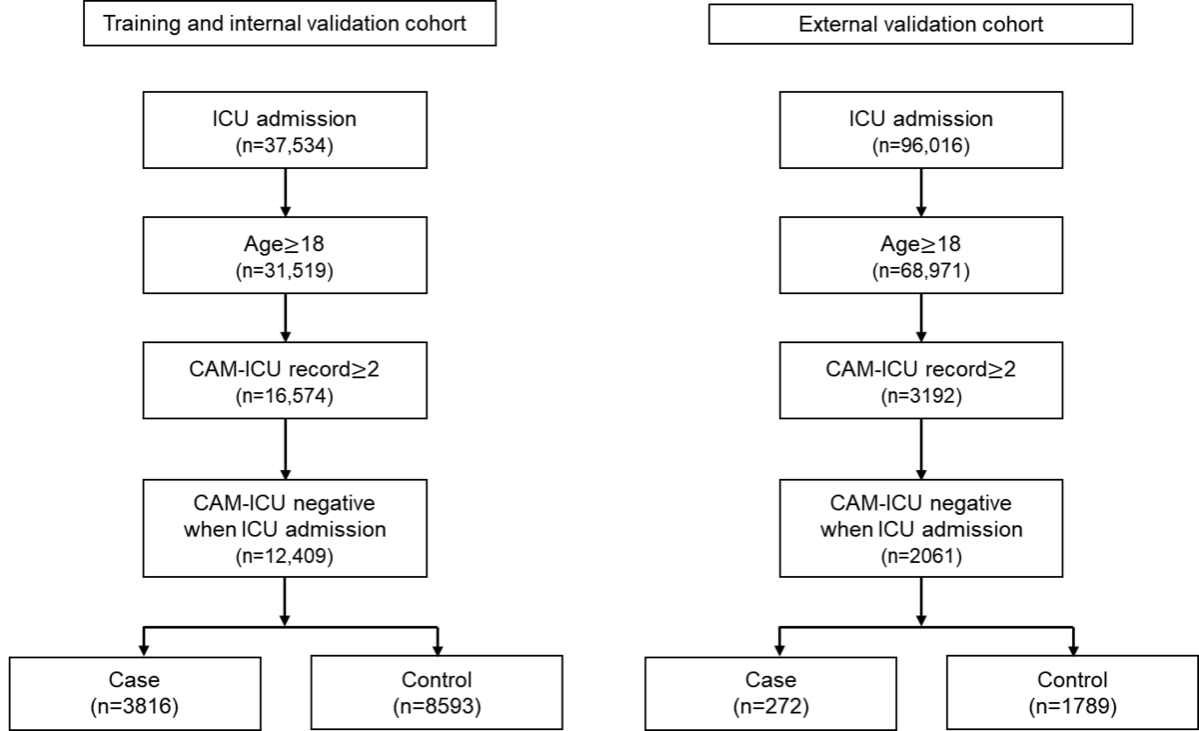
Flow diagram of the participant selection process. CAM-ICU: confusion assessment method for the intensive care unit; ICU: intensive care unit.

We conducted a retrospective study of all critically ill patients admitted to the ICUs of the Samsung Medical Center (a 1989-bed university-affiliated, tertiary referral hospital in Seoul, South Korea) from July 1, 2016, to August 31, 2019. We only included patients who were 18 years or older at the time of admission and who stayed in the medical or surgical ICU. Patients were excluded if they lacked a Confusion Assessment Method for the ICU (CAM-ICU) record from the day of ICU admission or if they had a positive CAM-ICU record at the time of ICU admission. The flow diagram in Figure 1 shows the patient selection process. The study protocol was removed from all identifiers and approved by the SMC (Samsung Medical Center) Institutional Review Board (IRB No. 2020-02-026), as all identifiers were removed. The IRB approval form is presented in Multimedia Appendix 1.

### Source of Data

This study used data from the Clinical Data Warehouse Darwin-C database of the SMC and the Medical Information Mart for Intensive Care III (MIMIC-III) database (v1.4). The SMC data set was used for the derivation and validation cohort, and the MIMIC-III data set was used for the external validation cohort. The MIMIC-III database is a clinical database consisting of data from more than 38,000 ICU patients (medical, surgical, trauma-surgical, coronary, and cardiac-surgery data) admitted to Beth Israel Deaconess Medical center (Boston, MA) from June 2001 to October 2012 [11]. The MIMIC-III database can be accessed upon obtaining approval from its administrators.

### Outcome

To screen for delirium, all ICU patients were assessed with the CAM-ICU [12]. The primary outcome of the study was the prediction of the occurrence of delirium within 24 hours of ICU admission. Delirium was defined as a negative CAM-ICU result obtained within the first 4 hours, and a positive CAM-ICU result obtained between 4 and 24 hours of ICU admission. In our institute, CAM-ICU results were obtained 3 times a day, and a senior nurse rechecked the recorded CAM-ICU scores.

### Predictor Variables

We used clinical characteristics, ICU admission category (medical or surgical), primary cause of admission (respiratory, cardiovascular, gastrointestinal, neurology, perioperative, nephrology, metabolic, or trauma), primary diagnosis, vital signs, prescription medications, and laboratory test results as the predictor variables. All variables were extracted from the EHR data set.

### Feature Selection and Data Processing

We first extracted all relevant variables for the prediction model from other studies. Next, 2 clinical experts (CRC and REK) reviewed the relevant variables and selected the crucial ones based on previous clinical studies and clinical relevance. We then further restricted the variables depending on whether they could be automatically extracted from EHRs and had low missing rates. Finally, for the external validation in the MIMIC-III data set, we selected variables found in both SMC and MIMIC-III. The MIMIC-III data set shows the final variables used as input for model development. The list of variables used is shown in Textbox 1, and the missing rate in the variable list is presented in Multimedia Appendix 2.

**Variables used for model development.**

**General information**

- Age, sex, and invasive mechanical ventilation

**Admission category**

- Medical intensive care unit (ICU) or surgical ICU

**Reason for ICU admission**

- Respiratory, cardiovascular, gastrointestinal, neurology, perioperative, nephrology, metabolic, and trauma

**Vital signs**

- Systolic blood pressure, diastolic blood pressure, heart rate, respiratory rate, peripheral capillary oxygen saturation, and Glasgow Coma Scale (eye, verbal, and motor)

**Comorbidity**

- Charlson Comorbidity Index

**Laboratory tests**

- Complete blood count:
  white blood count, hemoglobin, hematocrit, platelet count, and erythrocyte sedimentation rate
- Coagulation:
  prothrombin time (INR) and activated partial thromboplastin time
- Chemistry:
  Total protein, albumin, total bilirubin, aspartate aminotransferase, alanine aminotransferase, glucose fasting, blood urea nitrogen, creatinine, phosphorus, sodium, potassium, magnesium, calcium (ionized), C-reactive protein quantitative, and lactic acid
- Arterial Blood Gas Analysis:
  pH, PaCO_2_, PaO_2_, HCO_3_, and O_2_ Saturation

**Medications**

- Antibiotics, anticholinergic and antipsychotics, benzodiazepines, miscellaneous antidepressants, anxiolytics, sedatives and hypnotics, vasopressors, opiate agonists, opiate antagonists, cholinergic agents, and steroids

With regard to the general data processing, we first processed invalid values by eliminating them. Invalid values include extreme outliers in numerical values (for example, numerical values for vitals are eliminated using certain rules (ie, heart rate values should be between 0 and 300). Second, we processed numerical values by normalizing and scaling them. We performed standard normalization and min-max scaling such that the final numerical values were between 0 and 1. Finally, we processed the missing values. Missing values in the numerical data were filled with mean values, whereas missing values in categorical data were left blank such that the dummy variables were all equal to 0.

For certain variables with temporal information, such as vital values and laboratory test results, we determined statistical values such as the mean, standard deviation, min, max, and the closest values to the ICU admission to ensure multiple rows of numerical values can be summarized into one. Subsequently, to reduce the number of features necessary to train the model, we picked only one of the statistical values according to the feature importance of the random forest (RF). For example, there were initially multiple values for diastolic blood pressure (DBP) with respect to time. We calculated statistical values such as the mean, standard deviation, min, max, and the latest DBP values. Finally, we only selected the mean DBP because it was the most important among the statistical values of the DBP according to the feature importance of the RF.

### Model Development and Validation

We split the data set into a development data set and a data set. For the development data set, we used the data obtained between July 1, 2016, and December 31, 2018. For the validation set, we used the data obtained between January 1, 2019, and August 31, 2019. Of the 37,543 admitted cases, 12,409 cases were selected in this study. These were divided into the development set (n=9589, 77.3%) and the internal validation set (n=2820, 22.7%). Among the 9589 cases in the development data set, there were 3060 (31.9%) cases of delirium, and among the 2820 cases in the validation data set, there were 756 (26.8%) cases of delirium. We did not apply specific methodology (eg, undersampling) to resolve the outcome imbalance problem because it was not extreme.

We employed RF, extreme gradient boosting (XGBoost), deep neural network (DNN), and logistic regression (LR) as the candidate prediction models.

### Parameter Tuning

We also used an automated machine learning called the Tree-based Pipeline Optimization Tool for model selection and parameter searching [13].

For DNN, we used 512, 256, and 128 neurons for hidden layers, ReLU function for activation function in hidden layers, sigmoid function for activation function in the output layer, and binary cross-entropy function as the loss function. For XGBoost, we used a tree booster with 100 estimators, the learning rate as 0.1, and the subsample ratio as 0.75.

### External Validation

After development and internal validation, we performed the external validation of our delirium prediction model using the MIMIC-III database. The validation set was extracted from the MIMIC-III database, which included patients with at least two CAM-ICU records obtained within at least 24 hours.

The model with the highest area under the receiver operating characteristics (AUROC) curve in the external validation was named the PRIDE (Prediction of ICU Delirium) algorithm.

### Statistical Analysis

Continuous variables are presented in terms of means and SD, and categorical variables are presented in terms of their frequencies and percentages. The performances of the different models were compared using the AUROC, sensitivity, specificity, positive predictive value (PPV), and negative predictive value (NPV) at the threshold. In the internal validation, model performance was evaluated through the average and 95% CI of the AUROCs. Additionally, we used a calibration curve and the Brier score to test the reliability of our model. To determine the clinically relevant threshold, we used a decision curve.

We employed the TRIPOD (Transparent Reporting of a Multivariable Prediction Model for Individual Prognosis or Diagnosis) statement to report the results of our prediction model. Data processing, statistical analysis, and the development and validation of the machine learning algorithms were performed using R version 3.6.2 [14] and Python version 3.6.8 [15].

The source code has been made available on Github [16].

## Results

### Study Population

During the study period, a total of 37,543 cases were collected. Patients who were 18 years or older at the time of ICU admission were included. Cases with less than two CAM-ICU records after admission to the ICU and those with a positive CAM-ICU upon ICU admission were excluded. After patient exclusion, 12,409 remained as our study population. The case group consisted of 3816 (30.8%) patients who experienced delirium incidents during the study period. With regard to the MIMIC-III (external validation) data set, patients younger than 18 years of age, those with less than two CAM-ICU records recorded within 24 hours, and those with positive CAM-ICU records upon ICU admission were excluded. Based on the exclusion criteria, out of the 96,016 ICU admission cases, 2061 cases were included, and 272 (13.2%) delirium incidents occurred.

Baseline characteristics of the training and test sets of the SMC and MIMIC-III data sets are shown in Table 1.

**Table 1:** Baseline characteristics of data sets.

Characteristics			Development					Internal validation			External validation (MIMIC-III^a^)		
			Case (n=3060)		Control (n=6529)		Case (n=756)		Control (n=2064)	Case (n=272)			Control (n=1789)
Age (years), mean (SD)			65.4 (14.4)		61.1 (13.3)		65.3 (14.7)		59.7 (14.0)	82.5 (59.7)			73.7 (52.3)
Sex (male), n (%)			1994 (65.2)		4227 (64.7)		466 (61.6)		1190 (57.7)	137 (50.4)			925 (51.7)
**Admission category, n (%)**													
	Medical	1431 (46.8)		1639 (25.1)		293 (38.8)			344 (16.7)	151 (55.5)		1,141(63.8)	
	Surgical	1629 (53.2)		4890 (74.9)		1,720 (83.3)			463 (61.2)	121 (44.5)		648(36.2)	
**Reason for ICU^b^ admission, n (%)**													
	Respiratory	692 (22.6)		374 (5.7)		166 (22.0)			71 (3.4)	34 (12.5)		247 (13.8)	
	Cardiovascular	480 (15.7)		1166(17.9)		105 (13.9)			283 (13.7)	73 (26.8)		561 (31.4)	
	Gastrointestinal	145 (4.7)		139 (2.1)		28 (3.7)			25 (1.2)	55 (20.2)		379 (21.2)	
	Neurology	102 (3.3)		115 (1.8)		46 (6.1)			174 (8.4)	57 (21.0)		270 (15.1)	
	Peri-operation	1168 (38.2)		4278 (65.5)		320 (42.3)			1448 (70.2)	14 (5.1)		81 (4.5)	
	Nephrology	83 (2.7)		78 (1.2)		17 (2.2)			17 (0.8)	4 (1.5)		54 (3.0)	
	Metabolic	15 (0.5)		6 (0.1)		1 (0.1)			0 (0.0)	5 (1.8)		85 (4.8)	
	Hematology	15 (0.5)		22 (0.3)		4 (0.5)			6 (0.3)	5 (1.8)		33 (1.8)	
	Trauma	10 (0.3)		10 (0.2)		2 (0.3)			0 (0.0)	25 (9.2)		79 (4.4)	
	Others	350 (11.4)		341 (5.2)		67 (8.9)			40 (1.9)	—		—	
Initial SOFA^c^, mean (SD)			7.1 (3.6)		3.2 (2.7)		7.0 (3.7)		2.8 (2.5)	5.4 (3.3)			3.1 (2.4)
Vasopressor^d^, n (%)			1,145 (37.4)		793 (12.1)		292 (38.6)		175 (8.5)	38 (14.0)			71 (4.0)
Invasive mechanical ventilator, n (%)			1,900 (62.1)		1,165 (17.8)		456 (60.3)		344 (16.7)	19 (7.0)			73 (4.1)
CCI^e^, mean (SD)			0.9 (2.2)		0.3 (1.3)		1.1 (2.5)		0.4 (1.4)	3.2 (1.8)			2.8 (1.8)
**Comorbidity, n (%)**													
	Heart disease	149 (4.9)		123 (1.9)		17 (2.2)			20 (1.0)	33 (12.1)		232 (13.0)	
	Stroke	98 (3.2)		33 (0.5)		29 (3.8)			24 (1.2)	16 (5.9)		57 (3.2)	
	Malignancy	434 (14.2)		319 (4.9)		80 (10.6)			60 (2.9)	5 (1.8)		12 (0.7)	
	Renal failure	71 (2.3)		99 (1.5)		16 (2.1)			26 (1.3)	17 (12.1)		106 (12.7)	
	Liver disease	146 (4.8)		92 (1.4)		23 (3.0)			15 (0.7)	38 (14.0)		168 (9.4)	
	Dementia	55 (1.8)		9 (0.1)		17 (2.2)			6 (0.3)	—		—	
**Vital signs, mean (SD)**													
	Systolic BP^f^	125.8 (24.7)		127.4 (21.7)		128.6 (26.4)			130.8 (22.2)	118.5 (25.0)		119.5 (22.7)	
	Diastolic BP	71.6 (15.5)		74.3 (14.1)		72.3 (17.9)			73.3 (14.7)	63.3 (15.9)		63.9 (15.4)	
	Heart rate	85.4 (20.7)		80.7 (16.6)		84.5 (20.5)			80.6 (16.1)	89.9 (20.3)		85.8 (19.4)	
	Respiratory rate	19.3 (3.9)		18.3 (2.5)		18.8 (3.7)			17.7 (2.5)	20.5 (6.5)		19.2 (5.4)	
	SpO_2_^g^	96.1 (5.5)		96.9 (4.3)		96.5 (4.0)			97.4 (2.1)	95.9 (3.6)		96.2 (3.1)	
	Body temperature (°C)	36.7 (0.8)		36.6 (0.5)		36.7 (0.8)			36.6 (0.4)	36.8 (0.7)		36.8 (0.6)	
**ABGA^h^, mean (SD)**													
	pH	7.4 (0.1)		7.4 (0.1)		7.4 (0.1)			7.4 (0.1)	7.4 (0.1)		7.4 (0.1)	
	PaCO_2_	35.5 (14.1)		36.2 (7.5)		35.3 (11.3)			36.4 (6.2)	42.0 (12.4)		41.1 (12.1)	
	PaO_2_	119.8 (83.4)		184.4 (110.8)		122.0 (82.5)			190.4 (104.8)	131.9 (106.0)		125.0 (67.9)	
	HCO_3_	22.0 (5.5)		23.1 (3.6)		22.0 (5.3)			23.5 (2.9)	24.1 (5.4)		24.2 (4.7)	

^a^MIMIC-III: Medical Information Mart for Intensive Care III.

^b^ICU: intensive care unit.

^c^SOFA: sequential organ failure assessment.

^d^Vasopressor: epinephrine, norepinephrine, dobutamine, dopamine, vasopressin.

^e^CCI: Charlson Comorbidity Index.

^f^BP: blood pressure.

^g^SpO_2_: peripheral capillary oxygen saturation.

^h^ABGA: arterial blood gas analysis.

### Internal Validation

The average AUROCs and 95% CIs for internal validation were 0.919 (95% CI 0.919-0.919) for XGBoost, 0.916 (95% CI 0.916-0.916) for RF, 0.881 (95% CI 0.878-0.884) for DNN, and 0.875 (95% CI 0.875-0.875) for LR. For each model, we selected the highest value of specificity among sensitivities over 0.9 as the cut-off point for the threshold. The best model for the internal validation was XGBoost, with an AUROC of 0.919 (95% CI 0.919-0.919). Its sensitivity, specificity, PPV, and NPV were 0.904 (95% CI 0.904-0.905), 0.731 (95% CI 0.729-0.732), 0.565 (95% CI 0.563-0.566), and 0.952 (95% CI 0.952-0.952), respectively.

### External Validation

**Figure 2:**
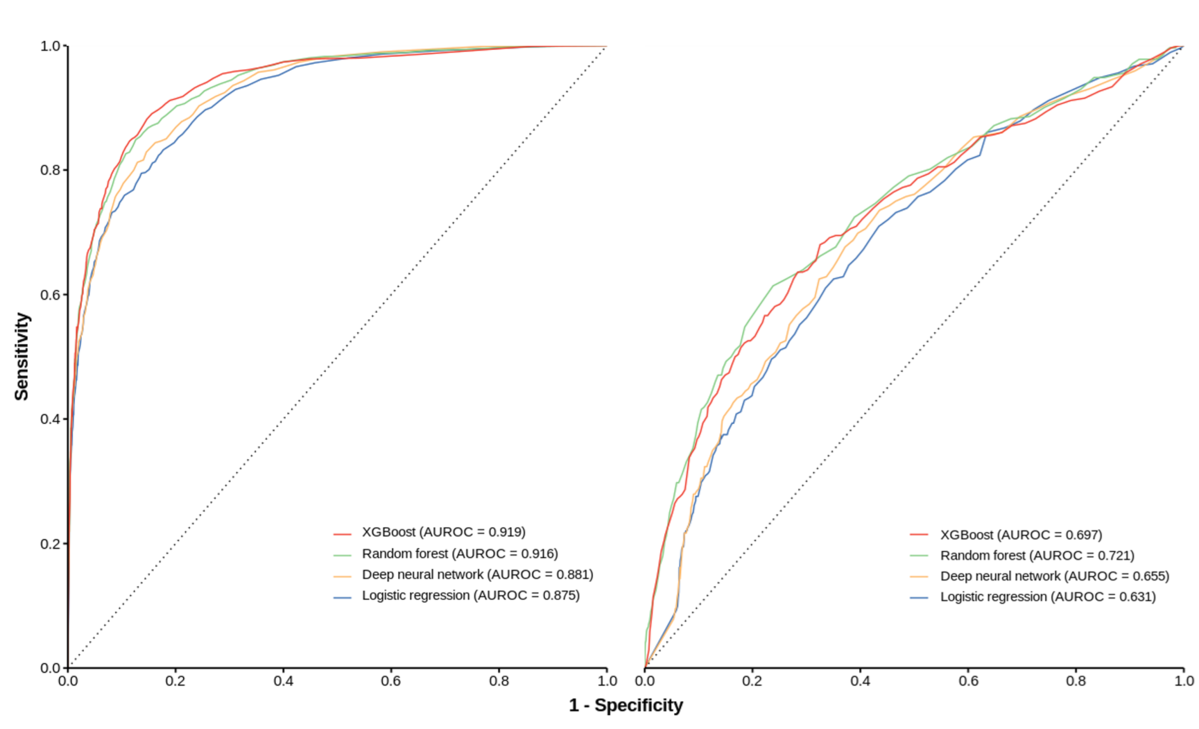
Receiver operating characteristic curves for all the prediction of intensive care unit delirium models. AUROC: area under the receiver operating characteristic curve; XGBoost: extreme gradient boosting.

For the external validation, the average AUROCs and 95% CI were 0.721 (95% CI 0.72-0.721) for RF, 0.697 (95% CI 0.695-0.699) for XGBoost, 0.655 (95% CI 0.654-0.657) for DNN, and 0.631 (95% CI 0.631-0.631) for LR. For the external validation on the MIMIC-III database, the model with the best AUROC was the RF model, with an AUROC of 0.721 and a sensitivity, specificity, PPV, and NPV of 0.91 (95% CI 0.909-0.912), 0.27 (95% CI 0.266-0.273), 0.159 (95% CI 0.159-0.16), and 0.952 (95% CI 0.951-0.953), respectively. A comparison of the performances of all of the models is shown in Table 2, and the ROC curves are shown in Figure 2.

**Figure 3:**
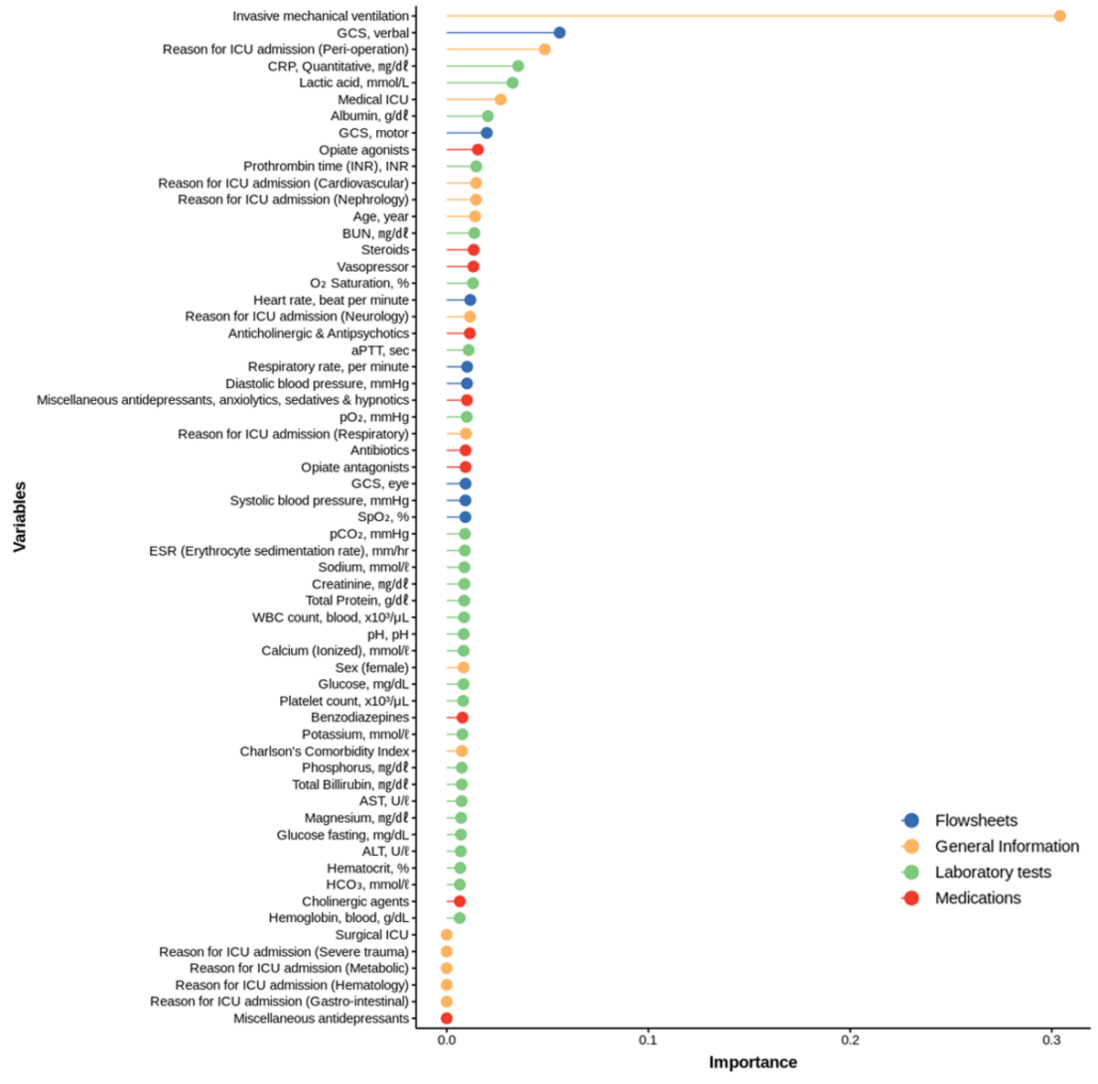
Variable importance of the prediction of intensive care unit delirium model. ALT: alanine aminotransferase; aPTT: activated partial thromboplastin time; AST: aspartate aminotransferase; BUN: blood urea nitrogen; CRP: C-reactive protein; GCS: Glasgow Coma Scale; ICU: intensive care unit; INR: international normalized ratio.

For the external validation with MIMIC-III, we only selected variables that could be found both in SMC and MIMIC-III. As a result, only 59 variables were selected. The variables were categorized into general information, flowsheet, laboratory test results, and prescription of medication. The most important variable was the use of invasive mechanical ventilation in the general information. The importance of each final variable used in model development is shown in Figure 3.

**Table 2:** Predictive performance of each model.

Model and data set		AUROC^a^, mean (95% CI)	Sensitivity, mean (95% CI)	Specificity, mean (95% CI)	Positive predictive value, mean (95% CI)	Negative predictive value, mean (95% CI)
**Random forest**						
	Internal data set	0.916 (0.916-0.916)	0.904 (0.904-0.905)	0.746 (0.744-0.747)	0.579 (0.578-0.580)	0.953 (0.952-0.953)
	External data set	0.721 (0.720-0.721)	0.910 (0.909-0.912)	0.270 (0.266-0.273)	0.159 (0.159-0.160)	0.952 (0.951-0.953)
**XGBoost^b^**						
	Internal data set	0.919 (0.919-0.919)	0.904 (0.904-0.905)	0.731 (0.729-0.732)	0.565 (0.563-0.566)	0.952 (0.952-0.952)
	External data set	0.697 (0.695-0.699)	0.908 (0.906-0.909)	0.250 (0.245-0.255)	0.156 (0.155-0.156)	0.946 (0.945-0.947)
**Deep neural network**						
	Internal data set	0.881 (0.878-0.884)	0.906 (0.905-0.907)	0.622 (0.608-0.635)	0.485 (0.477-0.492)	0.944 (0.943-0.945)
	External data set	0.655 (0.654-0.657)	0.907 (0.905-0.908)	0.197 (0.192-0.201)	0.147 (0.146-0.147)	0.932 (0.931-0.933)
**Logistic regression**						
	Internal data set	0.875 (0.875-0.875)	0.901 (0.901-0.901)	0.605 (0.605-0.605)	0.469 (0.469-0.469)	0.940 (0.940-0.940)
	External data set	0.631 (0.631-0.631)	0.904 (0.904-0.904)	0.155 (0.155-0.155)	0.140 (0.140-0.140)	0.914 (0.914-0.914)

^a^AUROC: area under the receiver operating characteristic curve.

^b^XGBoost: extreme gradient boosting.

### Model Assessment

For further model evaluation, calibration and decision curve analyses were performed. The Brier score for the XGBoost model with regard to predicting delirium was 0.094 for the internal validation data set, indicating that our model is reliable. The best model for external validation is RF, with the Brier score of 0.168. A Brier score of 0 indicates a perfect calibration, and the closest the value is to 0, the better model calibration. The calibration plot is shown in Multimedia Appendix 3. The decision curve analysis showed that the net benefit was useful for determining the threshold. For the PRIDE algorithm, the threshold for delirium prediction was selected as 0.13, and at this cut-off point, the net benefit was 0.234. The PRIDE model has a wide range of threshold probabilities and offers reasonable clinical applicability. The decision curve analysis is presented in Multimedia Appendix 4.

## Discussion

### Principal Results

We have demonstrated that the proposed delirium prediction model, which employs a machine learning algorithm with EHR data, can predict the development of delirium in medical and surgical ICU patients. In addition to our internal validation, we externally validated our findings using the MIMIC-III patient database. With the PRIDE model, we showed that delirium prediction models could be automated exclusively using risk factors derived from EHR data. The three main results of our study are as follows: (1) the model predicted delirium within the first 24 hours of ICU admission by only using data collected within the first 4 hours after ICU admission, (2) all variables were extracted from EMR data obtained from both medical and surgical intensive care patients, and (3) the model showed acceptable performance with regard to the external validation data set.

Among the various departments in a hospital, the incidence of delirium is the highest in the ICU, and it is well-documented that delirium occurs in 25% of critically ill adults in ICUs within the first 24 hours after admission [17-19]. This data shows that the early prediction of delirium upon initial ICU admission is crucial. Furthermore, the early prediction of the development of delirium can help clinicians make clinical decisions at an optimal time and provide preventive and personalized care with nondrug interventions for high-risk patients. Examples of such care are cognitive stimulation, orientation improvement, and early mobilization [20].

### Comparison With Prior Work

Owing to the prevalence of delirium in patients admitted to ICUs, the routine use of preventive measures for delirium is recommended. However, previous studies have shown that clinicians’ predictions of the development of delirium are less accurate than those of ICU delirium prediction models [7]. Thus, delirium prediction models developed using machine learning can support clinicians in the early recognition of delirium, thereby immensely benefiting patients at high risk of delirium [21]. Furthermore, although several risk prediction models have been proposed, they are based on the manual evaluation of individual risk factors, and thus, may be challenging to implement [7,22,23]. Hence, in practice, automated models are preferable and more feasible. For these reasons, the implementation of automated tools for predicting the risk of delirium development using data extracted from EHR would improve clinical practices with regard to ICU management. Furthermore, the EHR-based prediction model uses a pipeline that automatically extracts variables and calculates models containing enough variables.

Previous studies have used several risk factors for delirium in ICUs, including age, severity score, cause of admission, usage of sedative agents, and laboratory results. In contrast with previous studies, the PRIDE model includes several additional variables such as vital signs (heart rate and blood pressure) and medication information that is excluded from EHRs. These differences allow our model to predict delirium incidents within 4 hours of ICU admission only using EHR data. Further, the PRIDE model did not include a severity score because this can only be obtained after 24 hours of ICU admission; in addition, since this information is separate from EMR data, using a severity score would require further efforts by the clinician. A few reports have also presented EMR-based machine learning models to predict delirium [24,25]. Whereas the prediction models presented in these reports are for all hospital-admitted patients, in this study, we developed a versatile model specifically for ICU patients at risk of delirium.

The strength of our study is the EMR-driven model that was both internally and externally validated, using SMC and MIMIC-III data, respectively. Although our result showed lower accuracy with external data than internal data, this result can be improved if the missing rate of key features decreases. In the case of CAM-ICU, 96% was missing in MIMIC-III. In addition, a decrease in accuracy with an external database was not uncommon in literature [26]. For example, a study predicting serious bacterial infections among fevers in children reported that the AUC of the external data was 0.26 lower than the internal data [27].

In clinical settings, missing values occur for various reasons. To handle missing data, we used mean values in the numerical data. We left the missing values in the categorical data blank such that the dummy variables were all equal to 0 method. Recently, deep learning–based advanced techniques, such as long short-term memory and recurrent neural network, were also introduced to impute missing data, and by employing these methods, they could improve model performances [28]. When choosing a missing handling method, knowing the missing pattern can improve model performance and work better when applied to clinical applications.

### Limitations

There are potential limitations to our study that should be acknowledged. First, our study was retrospectively performed and validated. Prospective interventional studies are needed to verify the performance of the model and to reconfirm its clinical usefulness. Second, a selection bias might exist because we selected variables available in all cohorts, and this study was conducted in a retrospective manner. Furthermore, we excluded patients without CAM-ICU data (47% of the total number of ICU-admitted patients). In this regard, it should be noted that the purpose of this study was to develop a readily available model; therefore, we only selected the variables that could be used commonly in all cohorts. Finally, although the CAM-ICU tool is regarded as highly sensitive and specific to the detection of ICU delirium, it has critical limitations. As it only has binary labels, we cannot access the degree of delirium exacerbation. Furthermore, it is recorded in a “point-in-time” manner; thus, there may be some patients whose CAM-ICU tests were missed because they were completed outside the study’s time frame [29,30].

### Conclusions

We have developed and validated the delirium prediction model, which can predict the occurrence of delirium within 24 hours of ICU admission, using clinical data obtained in the first 4 hours after ICU admission. The PRIDE algorithm has acceptable AUCROC and sensitivity; thus, it has the potential to help advise ICU physicians and prevent ICU delirium.

## Appendix

Multimedia Appendix 1 Institutional review board approval form.

Multimedia Appendix 2 The missing rate in the variable list.

Multimedia Appendix 3 Calibration curve of the best model.

Multimedia Appendix 4 Decision curve of the best model. PRIDE: prediction of intensive care unit delirium model.

## Abbreviations

AUROC: area under the receiver operating characteristic curve
CAM-ICU: confusion assessment method for the intensive care unit
DBP: diastolic blood pressure
DNN: deep neural network
EHR: electronic health record
ICU: intensive care unit
IRB: institutional review board
LR: logistic regression
MIMIC-III: Medical Information Mart for Intensive Care III
NPV: negative predictive value
PRIDE: Prediction of ICU Delirium
PPV: positive predictive value
RF: random forest
SMC: Samsung Medical Center
XGBoost: extreme gradient boosting
