# Supplementary material for: A Machine Learning–Based Algorithm for the Prediction of Intensive Care Unit Delirium (PRIDE): Retrospective Study
Source: JMIR Med Inform. 2021 Jul 26;9(7):e23401. doi: 10.2196/23401 (PMC8367129; doi:10.2196/23401)
Supplement: Multimedia Appendix 1 [file medinform_v9i7e23401_app1.pdf]

NOTIFICATION LETTER
OF SMC IRB REVIEW SUMMARY

Investigator :	Cha, Wonchul, M.D.<br>Assistant Professor, Department of Emergency Medicine, SMC
SMC IRB File No. :	2020-02-026
Protocol Title :	Development of delirium prediction algorithm after hospitalization, using the initial admission data from ICU

☑ **Exempt** ☐ Not exempt

* Please append details or summarize reason(s) for determination below:

The objective of this research is to develop delirium prediction algorithm after hospitalization, using the initial admission data from ICU by collecting the data retrospectively. The initial admission data are properly de-identified by CDW (clinical data warehouse). Therefore, it meets the IRB exemption research category 3.

* Investigator does not need to submit continuing review or other IRB related reviews to the IRB.
* If investigator needs to revise research protocol which goes beyond IRB exemption research scope, he/she should re-submit the initial project to the IRB.

Date of Confirmation : Feb. 13, 2020

Institutional Review Board
Samsung Medical Center
#81, Irwon-Ro, Gangnam-Gu, Seoul, Korea, 135-710

APPROVED SAMSUNG 삼성서울병원 Institutional Review Board

CONTENTS OF IRB REVIEW

Investigator :	Cha, Wonchul, M.D.<br>Assistant Professor, Department of Emergency Medicine, SMC
SMC IRB File No. :	2020-02-026
Protocol Title :	Development of delirium prediction algorithm after hospitalization, using the initial admission data from ICU

DOCUMENT REVIEWED

1. Delirium Protocol_ver2.0 200206
   - Justification of ICF waivers

2. Delirium CRF ver.2.0(20.02.06)

3. Investigator Declaration Form _ver 4.0_200108

4. PI's CV

5. A Request Form for IRB Expedited Review_ver1.0 200206

6. Publication Plan

Issued by

Seil Jang
IRB Secretary for Administration
Samsung Medical Center

Aug. 27, 2020
Date

APPROVED
SAMSUNG
삼성서울병원
Institutional Review Board

IRB Information

Institution :	Samsung Medical Center (SMC)
Address :	#81 Irwon-ro, Gangnam-gu, Seoul, Korea
IRB President :	Prof. Hojoong Kim, M.D., Ph.D.<br>Dept. of Pulmonary and critical care medicine

SMC IRB abides by the guideline of ICH and GCP.

SMC IRB and the Institution are registered at Office for Human Research Protections (OHRP), U.S. Dept. of Health & Human Services, U.S.A..

Related SMC IRB registration and accreditation information is as follows.

＊ Identifier No. (U.S. OHRP)

Organization :	IORG0000545 - Samsung Med Ctr
Assurance :	FWA00002750
IRBs :	IRB #1 : IRB00000877
	IRB #2 : IRB00005489
	IRB #3 : IRB00005490
	IRB #4 : IRB00005491
	IRB #5 : IRB00006642
	IRB #6 : IRB00006643
	IRB #7 : IRB00008480
	IRB #8 : IRB00008481
	IRB #9 : IRB00011389
	IRB#10 : IRB00011390

＊ Samsung Medical Center has earned the accreditation from the Association for the Accreditation of Human Research Protection Programs, Inc. (AAHRPP) on June 16, 2006.

APPROVED
SAMSUNG
삼성서울병원
Institutional Review Board
