# Supplementary figures and images for: A Machine Learning–Based Algorithm for the Prediction of Intensive Care Unit Delirium (PRIDE): Retrospective Study
Source: JMIR Med Inform. 2021 Jul 26;9(7):e23401. doi: 10.2196/23401 (PMC8367129; doi:10.2196/23401)

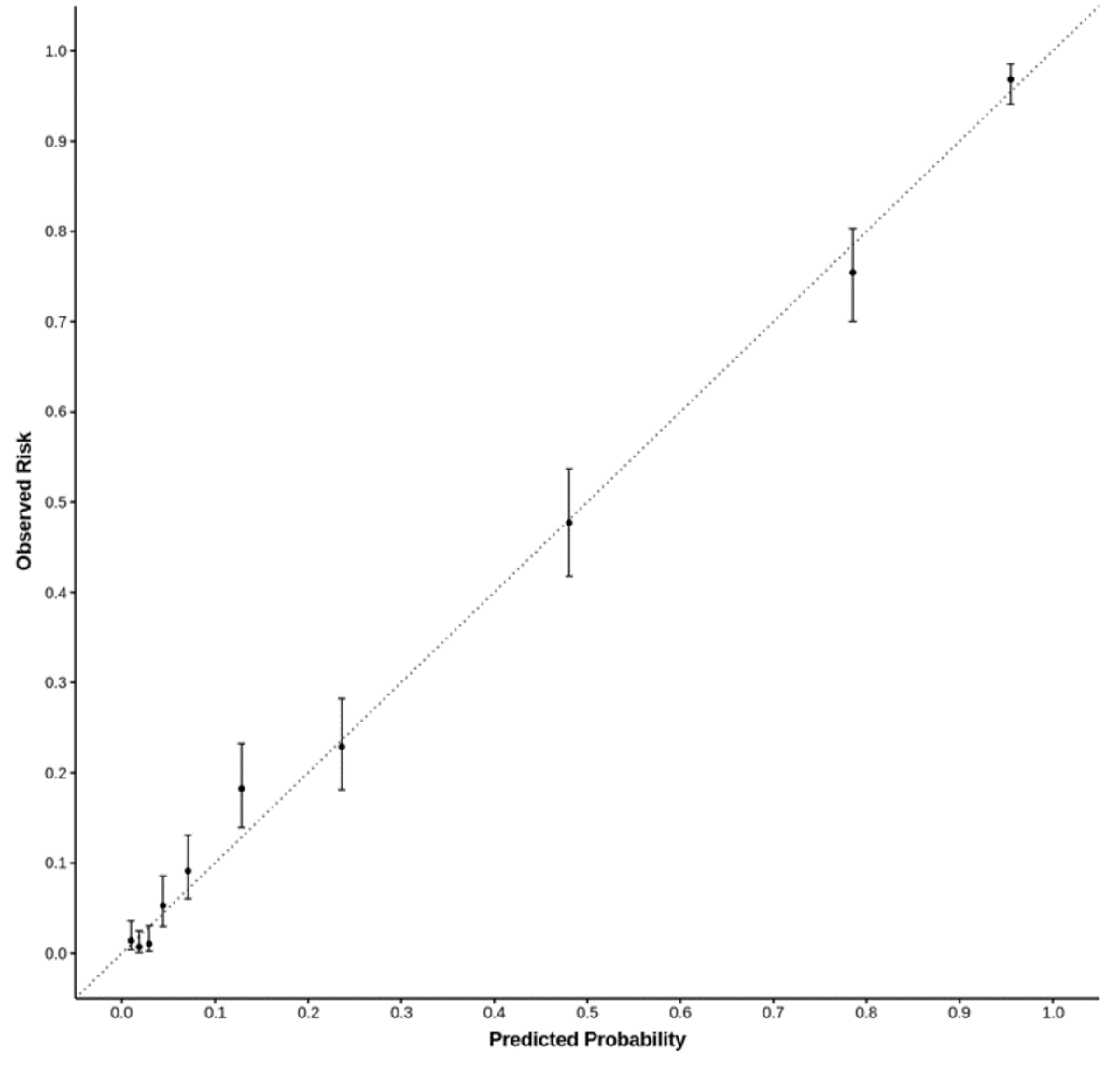

Supplement: Multimedia Appendix 3 [file medinform_v9i7e23401_app3.png]

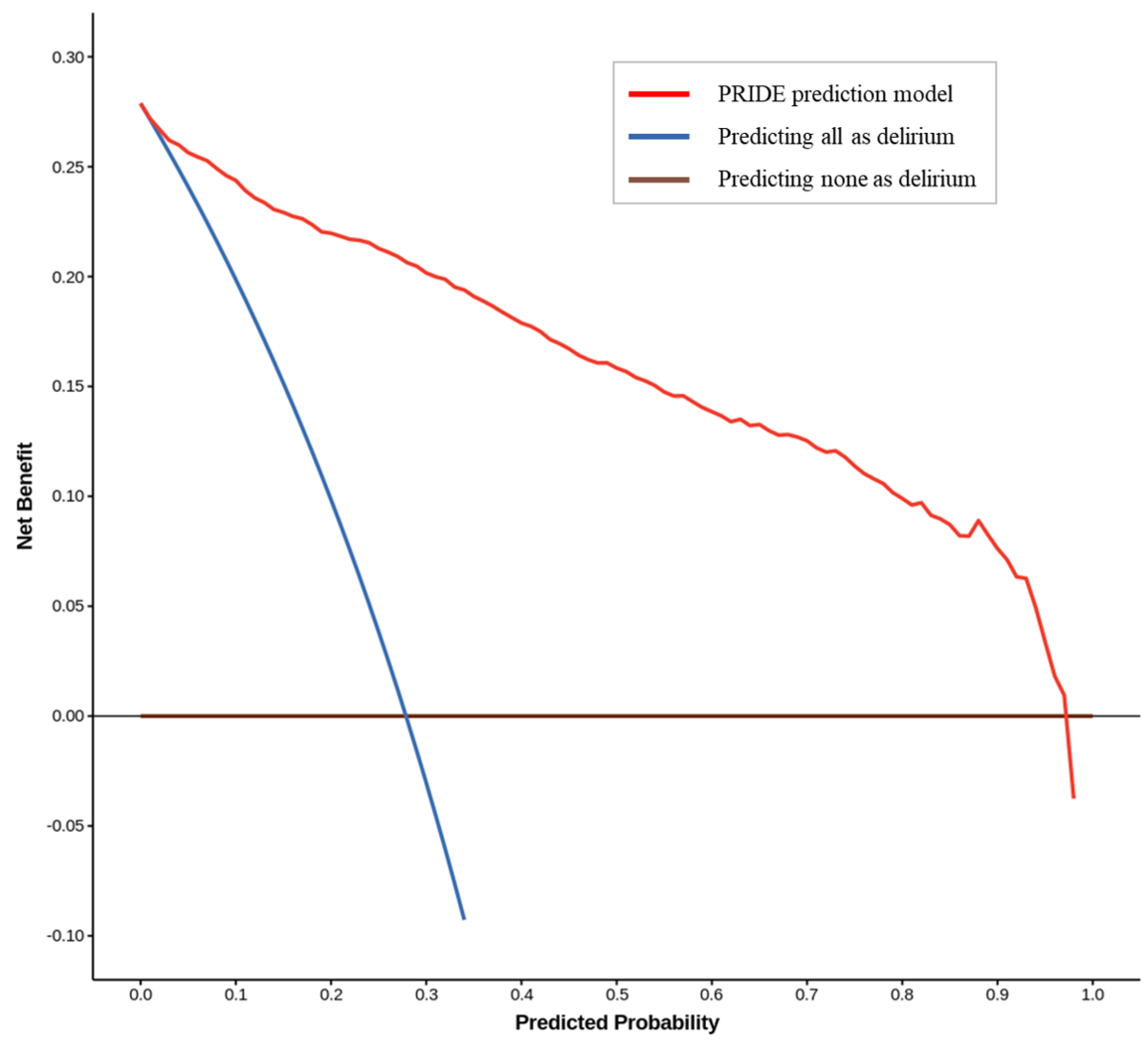

Supplement: Multimedia Appendix 4 [file medinform_v9i7e23401_app4.png]
